# HNRNPA2B1 promotes multiple myeloma progression by increasing AKT3 expression via m6A-dependent stabilization of ILF3 mRNA

**DOI:** 10.1186/s13045-021-01066-6

**Published:** 2021-04-01

**Authors:** Fengjie Jiang, Xiaozhu Tang, Chao Tang, Zhen Hua, Mengying Ke, Chen Wang, Jiamin Zhao, Shengyao Gao, Artur Jurczyszyn, Siegfried Janz, Meral Beksac, Fenghuang Zhan, Chunyan Gu, Ye Yang

**Affiliations:** 1 The Third Affiliated Hospital of Nanjing University of Chinese Medicine, Nanjing, 210001 China; 2 School of Medicine and Holistic Integrative Medicine, Nanjing University of Chinese Medicine, 138 Xianlin Road, Nanjing, 210023 China; 3 Department of Hematology, Jagiellonian University Medical College, Cracow, Poland; 4 Division of Hematology and Oncology, Medical College of Wisconsin, Milwaukee, USA; 5 Department of Hematology, School of Medicine, Ankara University, Ankara, Turkey; 6 Internal Medicine, University of Iowa, Iowa City, USA; 7 Myeloma Center, University of Arkansas, Little Rock, USA

**Keywords:** M6A, HNRNPA2B1, Multiple myeloma, MeRIP-Seq, ILF3, RNA stability, RIP-seq, AKT3

## Abstract

N6-methyladenosine (m6A) modification is the most prevalent modification in eukaryotic RNAs while accumulating studies suggest that m6A aberrant expression plays an important role in cancer. HNRNPA2B1 is a m6A reader which binds to nascent RNA and thus affects a perplexing array of RNA metabolism exquisitely. Despite unveiled facets that HNRNPA2B1 is deregulated in several tumors and facilitates tumor growth, a clear role of HNRNPA2B1 in multiple myeloma (MM) remains elusive. Herein, we analyzed the function and the regulatory mechanism of HNRNPA2B1 in MM. We found that HNRNPA2B1 was elevated in MM patients and negatively correlated with favorable prognosis. The depletion of HNRNPA2B1 in MM cells inhibited cell proliferation and induced apoptosis. On the contrary, the overexpression of HNRNPA2B1 promoted cell proliferation in vitro and in vivo. Mechanistic studies revealed that HNRNPA2B1 recognized the m6A sites of ILF3 and enhanced the stability of ILF3 mRNA transcripts, while AKT3 downregulation by siRNA abrogated the cellular proliferation induced by HNRNPA2B1 overexpression. Additionally, the expression of HNRNPA2B1, ILF3 and AKT3 was positively associated with each other in MM tissues tested by immunohistochemistry. In summary, our study highlights that HNRNPA2B1 potentially acts as a therapeutic target of MM through regulating AKT3 expression mediated by ILF3-dependent pattern.

**Supplementary Information:**

The online version contains supplementary material available at 10.1186/s13045-021-01066-6.

To the Editor

N6-methyladenosine (m6A) modification is the most frequent RNA modifications in eukaryotic RNAs affecting gene expression, which is seldom investigated in MM [1–3]. Therefore, we checked the m6A genes in MM compared to normal plasma cells and the correlation of these genes with patient outcome including HNRNPA2B1, Mettl3, Mettl14, Wtap, etc., in MM patient cohorts. Interestingly, HNRNPA2B1 was the exclusive gene, which was not only increased in MM samples but also associated with poor outcome in APEX, TT2 and HOVON65 patient cohorts (Fig. 1a, b, Additional file 1: Fig. S1a–h). HNRNPA2B1, RNA binding protein heterogeneous nuclear ribonucleoprotein A2B1, is a nuclear reader of m6A [4] and highly expressed in several cancers regulating the progression of cancer [5, 6] through multiple processes of mRNAs metabolism [7] including alternative splicing [8], cytoplasmic RNA trafficking [9], transcription and translation [10]. Here, we aimed to explore the potential functions and regulatory mechanism of HNRNPA2B1 in MM.

**Fig. 1:**
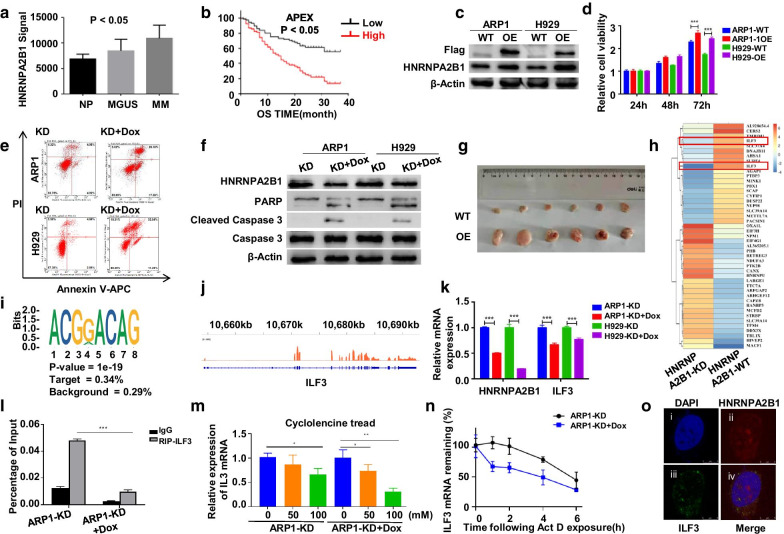
HNRNPA2B1 promotes MM proliferation and increases ILF3 protein expression through recognizing m6A modification and maintaining stabilization of ILF3 mRNA. **a** HNRNPA2B1 mRNA levels were significantly increased in MM samples. The signal level of HNRNPA2B1 was shown on the y-axis. Patients were designated as healthy donors with normal bone marrow plasma cells (NP, *n* = 22), monoclonal gammopathy of undetermined significance (MGUS, *n* = 44), or multiple myeloma (MM, *n* = 351), which were sorted on the *x*-axis. **b** Elevated HNRNPA2B1 mRNA was associated with poor overall survival (OS) in MM patients from the APEX patient cohort. **c** Overexpression of HNRNPA2B1 was confirmed by Western blotting after lentivirus infection in MM cells. **d** Overexpression of HNRNPA2B1 promoted cell proliferation in ARP1 and H929 cells. **e** Flow cytometry showed that HNRNPA2B1 inhibited MM cells apoptosis. **f** Expression of HNRNPA2B1 and apoptosis-related proteins in HNRNPA2B1^KD^ MM cells. **g** Tumor tissues were dissected from NOD-SCID mice injected with wild-type and HNRNPA2B1^OE^ cells. **h** Hot map of 44 differentially expressed genes (*P* < 0.05) in both m6A and transcription in ARP1 HNRNPA2B1^KD^ cells compared with controls. **i** HORMER motif analysis of m6A peaks in ARP1 and H929 cells. "Target" showed the percentage of peaks containing the identified consensus motif, "Background" presented the percentage of genome background regions that contain the identified motif. **j** IGV tracks depicted the position of m6A modification of ILF3 gene. **k** HNRNPA2B1 gene knockdown affected expression of ILF3 mRNA in MM cells. **l** RIP-qPCR assay was used to determine the interaction between HNRNPA2B1 and ILF3 mRNA in ARP1 cells. **m** The expression of ILF3 RNA was detected in ARP1 cells upon treating with cyclolencine in the concentration of 0 mM, 50 mM and 100 mM. **n** RT-qPCR following the addition of Actinomycin D (5 μg/mL) was performed to detect ILF3 mRNA stability. **k** HNRNPA2B1 (red) localization was examined by confocal microscopy. (i) Nuclei were stained with DAPI. Scale bar, 5 μm. (ii) Immunolocalization of HNRNPA2B1 in MM cells. (iii) Immunolocalization of ILF3. (iv) Merger of images of i, ii and iii, with the colocalized regions shown in orange. (*P < 0.05, **P < 0.01, ***P < 0.001)

Initially, we performed MTT assay that demonstrated the cellular proliferation was significantly increased in HNRNPA2B1 overexpression (HNRNPA2B1^OE^) cells and decreased in HNRNPA2B1 knockdown (HNRNPA2B1^KD^) cells (Fig. 1c, d, Additional file 1: Fig. S1i). Flow cytometry and WB analyses illustrated that knockdown of HNRNPA2B1 promoted cellular apoptosis (Fig. 1e, f). The nude mouse xenograft model by subcutaneous injection of HNRNPA2B1^OE^ cells showed overexpression of HNRNPA2B1 accelerating tumor growth in vivo (Fig. 1g and Additional file 1: Fig. S1j, k, l). These data indicated that HNRNPA2B1 promoted MM cellular growth in vitro and in vivo.

Next, m6A IP and RNA-seq analyses (MeRIP-Seq) found that ILF3 was downregulated (*P* < 0.05) of both m6A and transcription in HNRNPA2B1^KD^ cells compared with controls (Fig. 1h & Additional file 1: Fig. S2). The m6A consensus sequence (RRACH) motif [11] is shown in Fig. 1i, in which the m6A modification of ILF3 was enriched in 3′-noncoding regions (Fig. 1j). Consistently, ILF3 expression was reduced in HNRNPA2B1^KD^ cells (Fig. 1k, Additional file 1: Fig. S3a) while increased in HNRNPA2B1^OE^ cells (Additional file 1: Fig. S3b, c). RNA immunoprecipitation (RIP)-qPCR assay revealed that ILF3 mRNA was enriched in the precipitates of HNRNPA2B1 antibody and silencing of HNRNPA2B1 decreased the abundance of the ILF3 transcript binding to HNRNPA2B1 (Fig. 1l). To further verify the role of m6A in regulation of ILF3 expression, methylation inhibitor cyclolencine was used that induced remarkable reduction of ILF3 in MM cells (Fig. 1m, Fig. S3d, e). Therefore, we can conclude that HNRNPA2B1 may stabilize ILF3 mRNA to play an important role. As expected, the stability of ILF3 was decreased in HNRNPA2B1^KD^ cells (Fig. 1n, Additional file 1: Fig. S3f). The biological effects of HNRNPA2B1 might be related to its nucleocytoplasmic localization, as HNRNPA2B1 was distributed in both nuclear and cytoplasmic along with increased cytoplasmic localization of ILF3 (Fig. 1o, Additional file 1: Fig. S3g). The above results illustrated that HNRNPA2B1-induced expression of ILF3 was due to the enhanced stability of ILF3 mRNA transcripts upon recognition and bound of the m6A sites to HNRNPA2B1.

Notably, ILF3 expression was significantly elevated in plasma cells from MM patients (Fig. 2a) and associated with poor survival (Fig. 2b, Additional file 1: Fig. S4a, b). We established the ILF3 knockdown (ILF3^KD^) cells (Fig. 2c), which displayed decreased cell growth rate after induction compared to the non-induced cells (Fig. 2d). Apoptosis assay showed that ILF3 inhibited MM cellular apoptosis (Additional file 1: Fig. S4c, d, e). In addition, MTT result demonstrated that ILF3 knockdown by siRNA could reverse the cellular proliferation induced by increased HNRNPA2B1 suggesting that ILF3 is one of the most important m6A/HNRNPA2B1 targets in MM (Additional file 1: Fig. S4f, g).

**Fig. 2:**
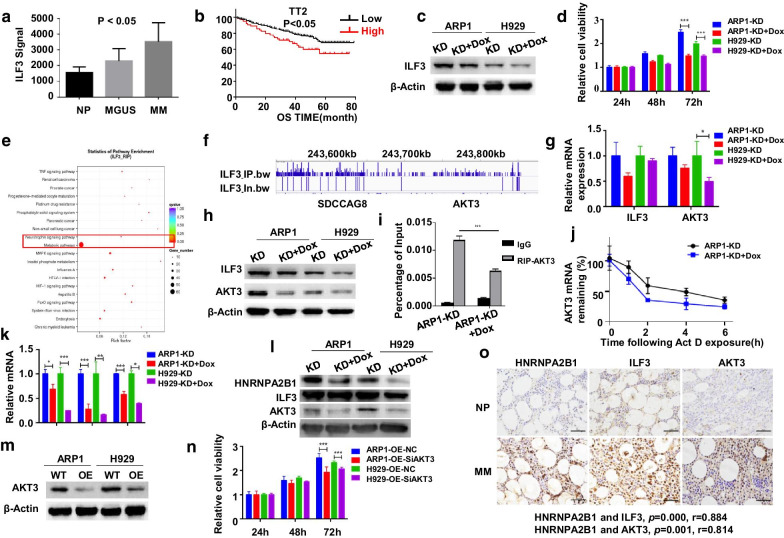
HNRNPA2B1 facilitates MM progression via enhancing ILF3-mediated expression of AKT3 in vitro. **a** ILF3 mRNA levels were significantly increased in MM samples. The signal level of ILF3 was shown on the y-axis. Patients were designated as healthy donors with normal bone marrow plasma cells (NP, *n* = 22), monoclonal gammopathy of undetermined significance (MGUS, *n* = 44), or multiple myeloma (MM, *n* = 351), which were sorted on the x-axis. **b** Increased ILF3 mRNA was associated with poor overall survival (OS) in MM patients from the APEX patient cohort. **c** The shRNA-mediated ILF3 repression was confirmed by Western blotting after lentivirus infection in ARP1 and H929 cells. **d** Effect of ILF3 knockdown on cell proliferation in MM cells. **e** The top 20 enriched KEGG pathways of the RIP-seq were presented as scatter plot. **f** Visualization of RIP-seq signal surrounding the AKT3 locus. **g** and **h** AKT3 expression under ILF3 silence was detected by RT-qPCR (**g**) and Western blotting (**h**). **i** RIP-qPCR assay was used to determine the interaction between ILF3 and AKT3 mRNA. **j** RT-qPCR following the addition of ActD (5 μg/mL) was used to detect AKT3 mRNA stability in ARP1 cells. **k** HNRNPA2B1 knockdown affected the expression of ILF3, AKT3 at mRNA level in ARP1 and H929 cells. **l** HNRNPA2B1 knockdown affected the expression of ILF3, AKT3 at protein level in ARP1 and H929 cells. **m** Protein expression was examined after HNRNPA2B1^OE^ cells treated with AKT3 small interfering RNA. **n** MTT assay indicated that targeting AKT3 by siRNA could reverse MM cell proliferation induced by HNRNPA2B1 overexpression. **o** Immunohistochemistry staining of HNRNPA2B1, ILF3 and AKT3 in primary MM samples (*n* = 12) and normal control (NP) (*n* = 12). (*P < 0.05, **P < 0.01, ***P < 0.001)

Further RNA immunoprecipitation-sequencing (RIP-seq) analysis indicated that MAPK pathway was enriched (Fig. 2e) and AKT3 mRNA was significantly enriched by anti-ILF3 antibody in ARP1 cells. The ILF3 binding sites represented by peaks were enriched in AKT3 mRNA transcripts (Fig. 2f). In agreement with above results, decreased AKT3 was observed in ILF3^KD^ cells (Fig. 2g, h) and RIP-qPCR using anti-ILF3 antibody confirmed a significantly reduced affinity of ILF3 to AKT3 mRNA in ARP1 ILF3^KD^ cells compared to control (Fig. 2i). RNA decay assay showed a relatively lower stability of AKT3 transcripts in ILF3^KD^ cells (Fig. 2j, Additional file 1: Fig S5a) correspondingly. These data suggested that ILF3 promoted MM progression through stabilization of AKT3 transcripts.

Finally, we verified the effect of HNRNPA2B1 on AKT3. The expression of AKT3 was decreased in HNRNPA2B1^KD^ cells (Fig. 2k, l), whereas the elevated expression of AKT3 was observed in HNRNPA2B1^OE^ cells (Additional file 1: Fig. S5b, c). While AKT3 was interfered with siRNA (Fig. 2m), the cellular proliferation induced by HNRNPA2B1 was attenuated (Fig. 2n). Intriguingly, immunohistochemistry correlation analysis showed that HNRNPA2B1, ILF3 and AKT3 were highly increased in MM patients with statistical-correlated expression trend significantly compared to normal controls (Fig. 2o).

In summary, we demonstrate the m6A-dependent effect of HNRNPA2B1 on regulating AKT signaling pathway and the correlation between HNRNPA2B1 and MM cell growth. It is disclosed that the HNRNPA2B1/m6A/ILF3/AKT3 axis plays a key role in MM progression.

## Supplementary Information

**Additional file 1**. HNRNPA2B1 is a high-risk MM marker and promotes MM progression via enhancing ILF3-mediated expression of AKT3 in vitro and in vivo.

**Additional file 2**. Detailed materials and methods.

**Additional file 3**.

## Data Availability

All supporting data are included in the manuscript and supplemental files. Additional data are available upon reasonable request to the corresponding author.

## Abbreviations

m6A: N6-methyladenosine
HNRNPA2B1: Heterogeneous nuclear ribonucleoprotein A2B1
MM: Multiple myeloma
MeRIP-Seq: M6A IP seqing
ILF3: Interleukin enhancer-binding factor 3
RIP: RNA immunoprecipitation
AKT3: AKT serine/threonine kinase 3
ActD: Actinomycin D
