# Supplementary figures and images for: HNRNPA2B1 promotes multiple myeloma progression by increasing AKT3 expression via m6A-dependent stabilization of ILF3 mRNA
Source: J Hematol Oncol. 2021 Apr 1;14:54. doi: 10.1186/s13045-021-01066-6 (PMC8017865; doi:10.1186/s13045-021-01066-6)

**Figure S1**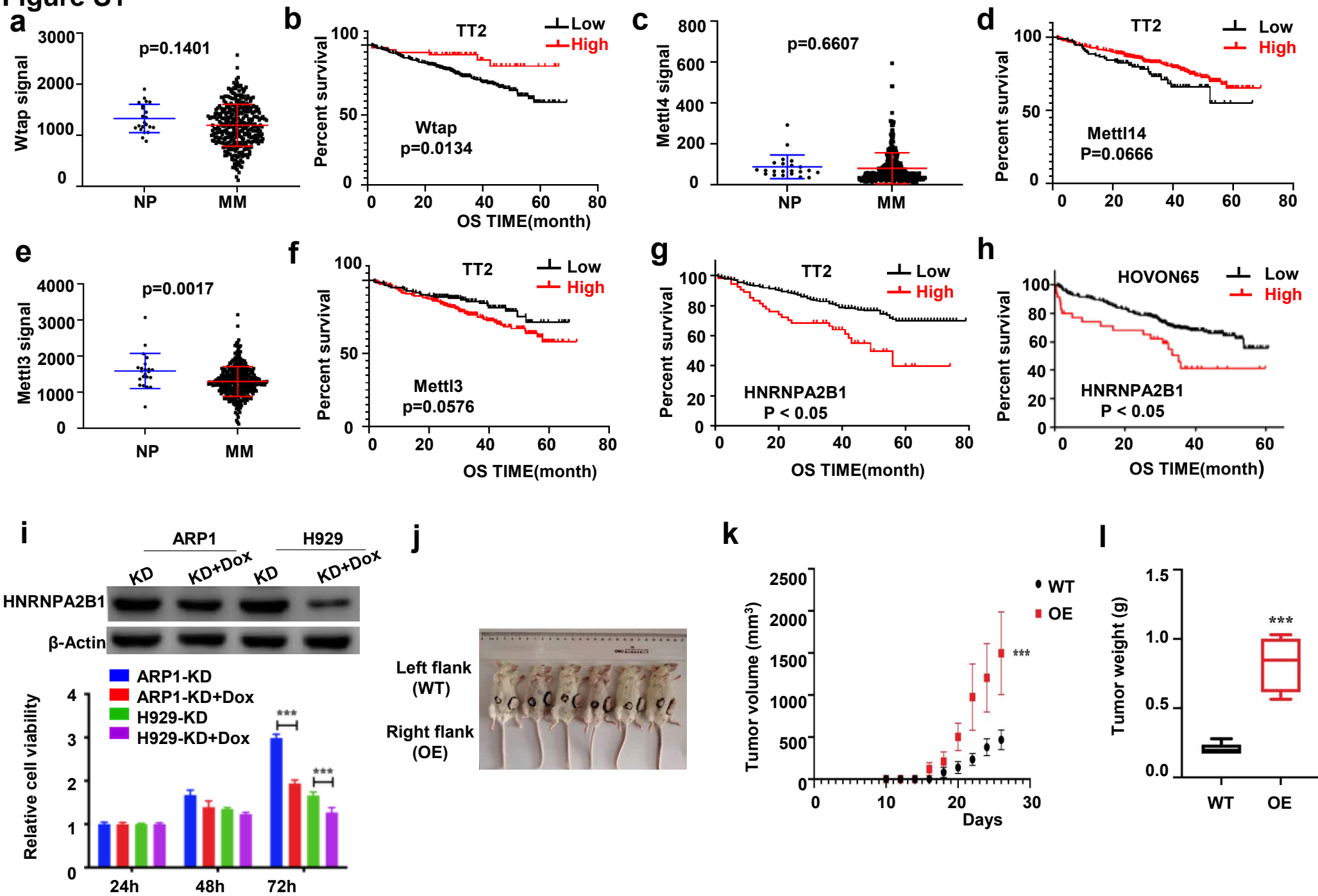

**Figure S2**

**a**

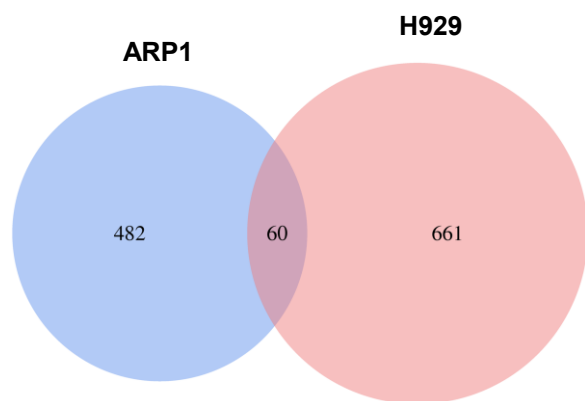

**b**

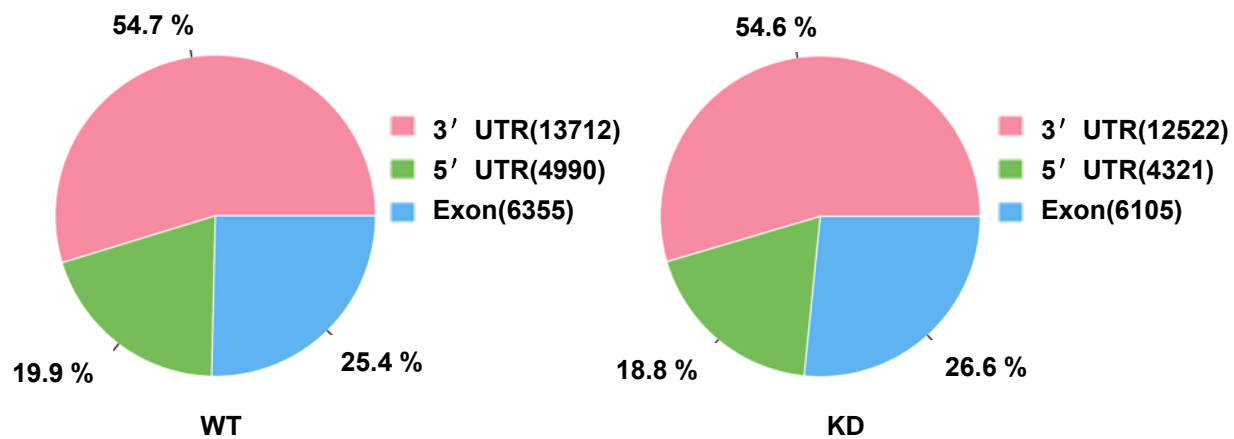

**c**

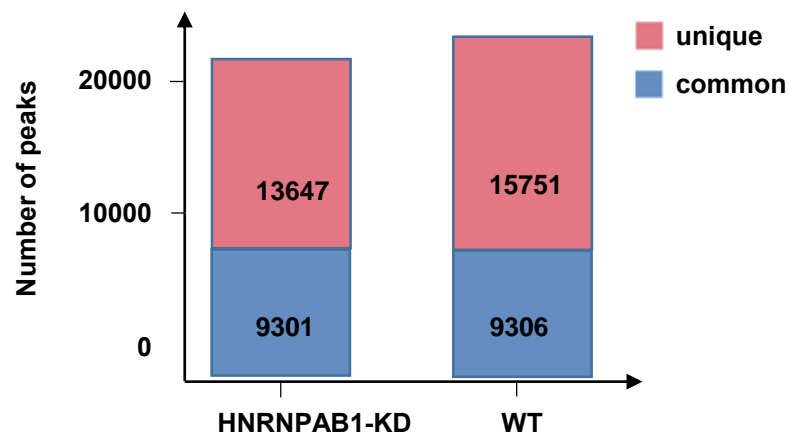

**d**

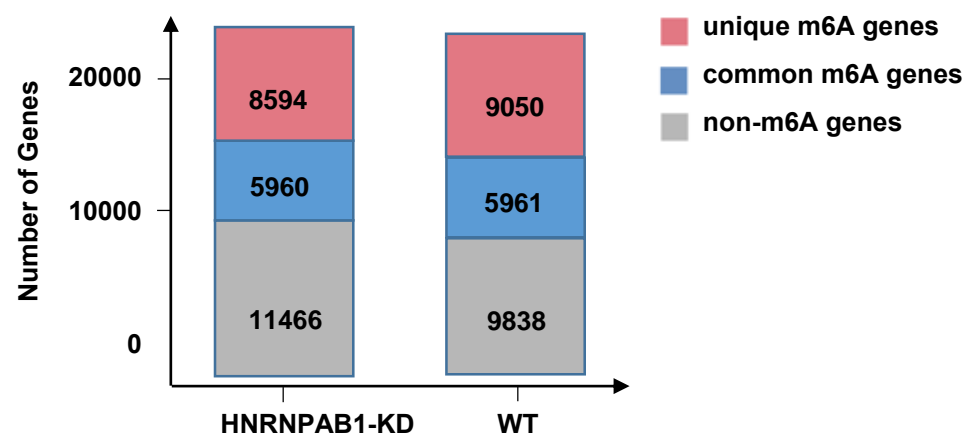

**Figure S3**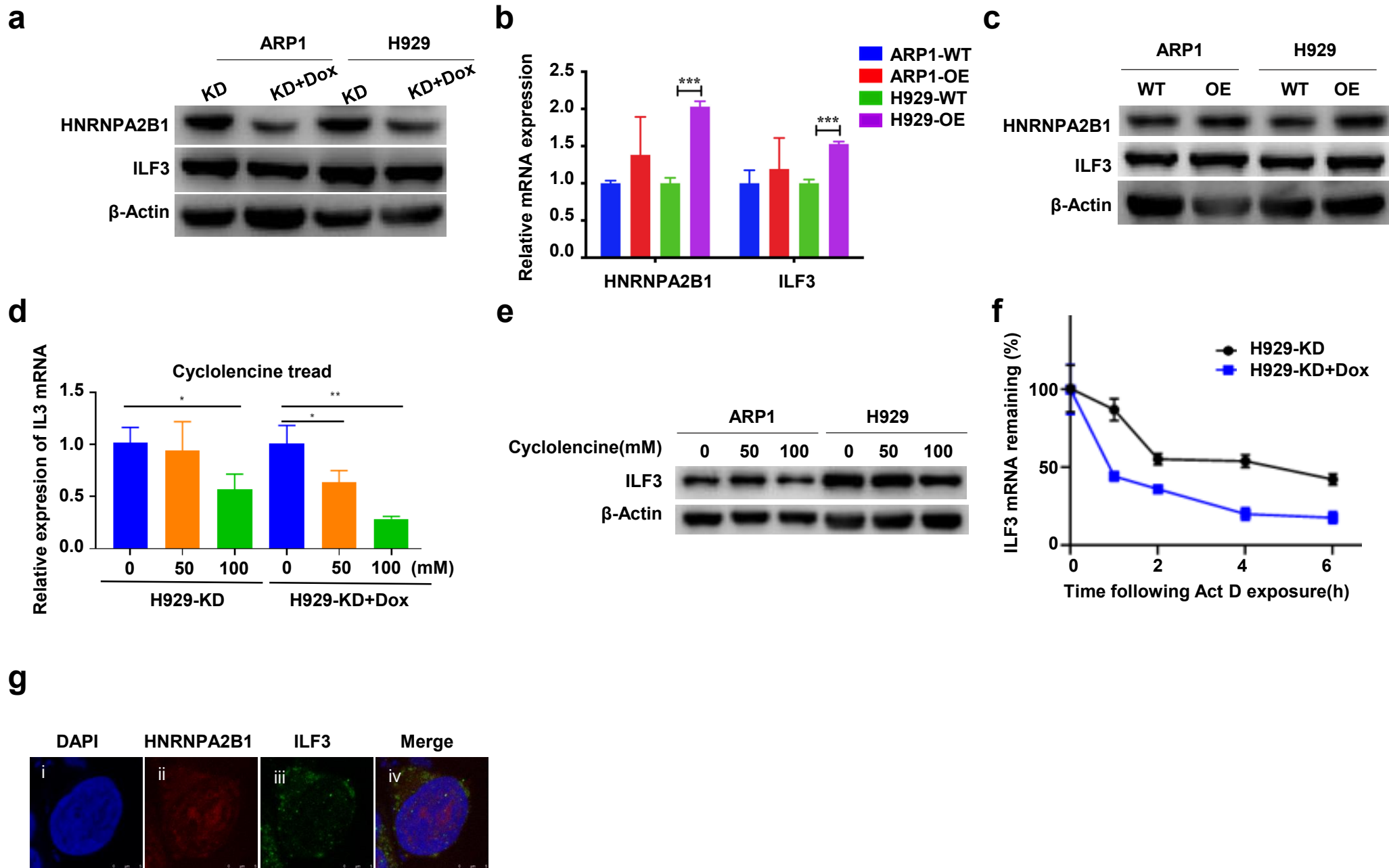

**Figure S4**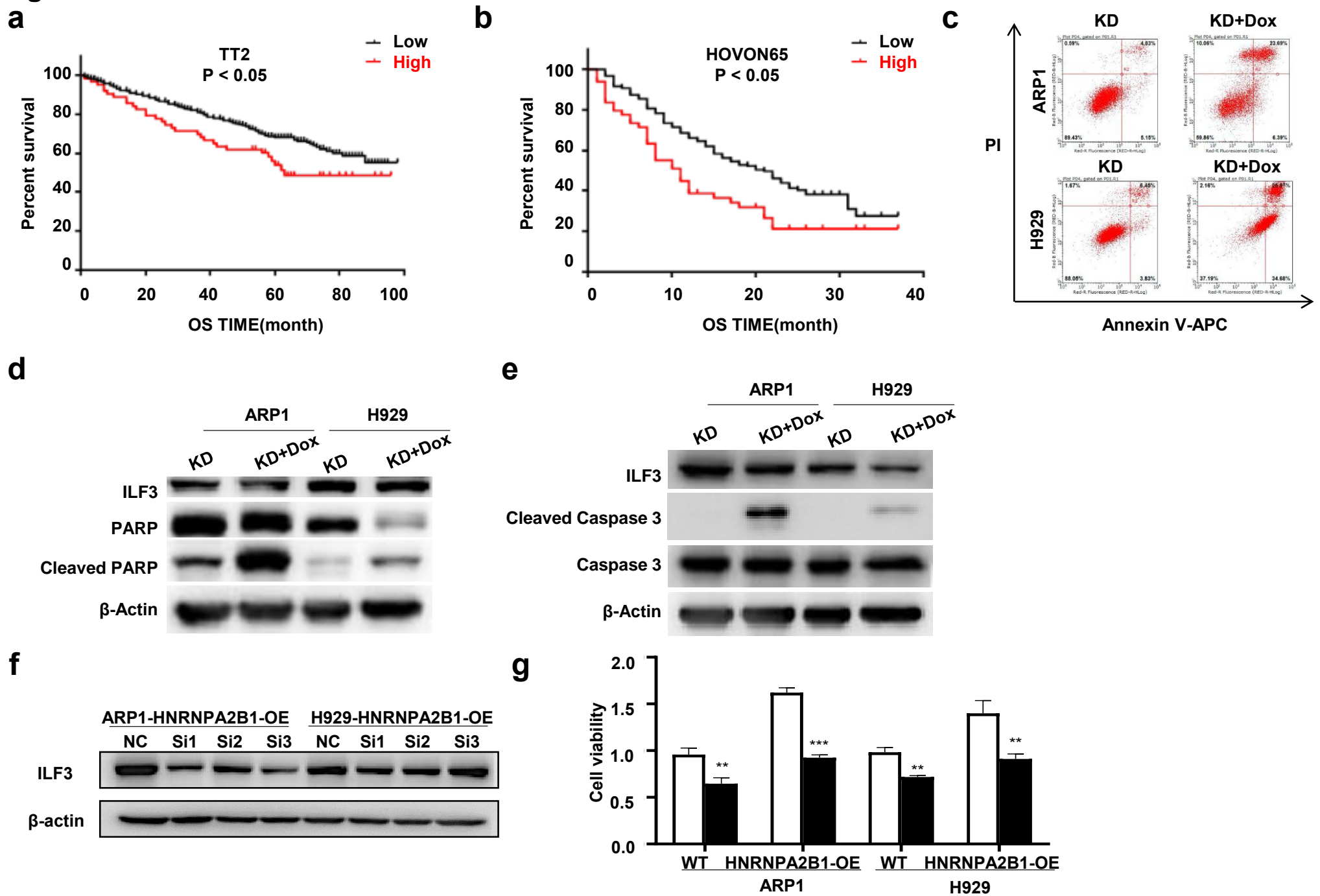

**Figure S5**

**a**

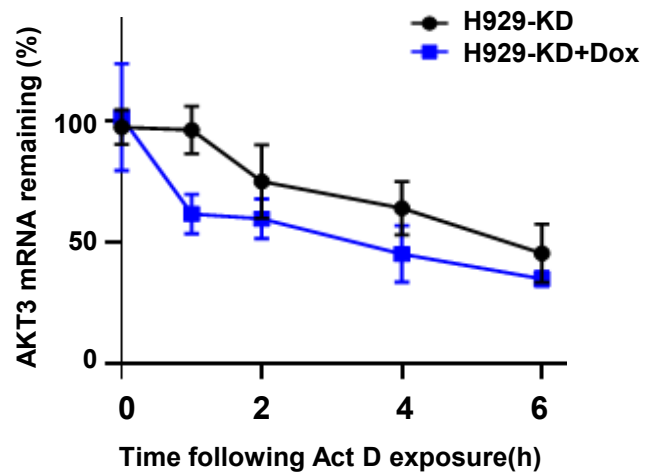

**b**

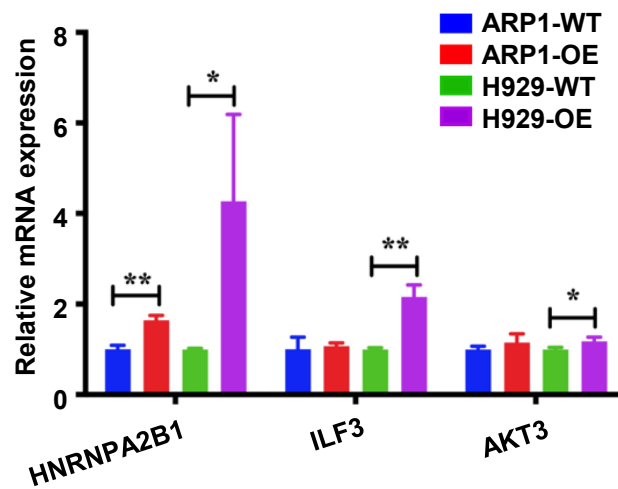

**c**

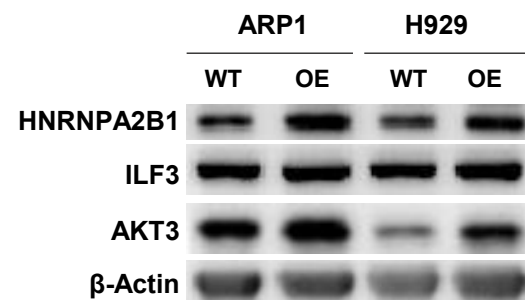

Supplement: Supplementary file 1 — Additional file 1. HNRNPA2B1 is a high-risk MM marker and promotes MM progression via enhancing ILF3-mediated expression of AKT3 in vitro and in vivo. [file 13045_2021_1066_MOESM1_ESM.pdf]
