# Supplementary material for: HNRNPA2B1 promotes multiple myeloma progression by increasing AKT3 expression via m6A-dependent stabilization of ILF3 mRNA
Source: J Hematol Oncol. 2021 Apr 1;14:54. doi: 10.1186/s13045-021-01066-6 (PMC8017865; doi:10.1186/s13045-021-01066-6)
Supplement: Supplementary file 2 — Additional file 2. Detailed materials and methods. [file 13045_2021_1066_MOESM2_ESM.docx]

**Additional file 1** Supplementary Figures present that *HNRNPA2B1* is a high-risk marker in MM and promotes MM progression via enhancing of *ILF3*-mediated expression of *AKT3* *in vitro* and *in vivo*.

**Figure S1.** *HNRNPA2B1* is a high-risk marker in MM. **a.** Wtap mRNA was not highly expressed in MM samples (p=0.1401). **b.** Decreased Wtap mRNA expression was associated with poor overall survival (OS) in MM patients from TT2 cohort (p=0.0134). **c.** Mettl14 mRNA was not increased in MM samples (p=0.6607). **d.** Increased Mettl14 mRNA expression was not related to poor overall survival (OS) in MM patients from TT2 cohort (p=0.0666). **e.** Mettl3 mRNA levels were significantly decreased in MM samples (p=0.0017). **f.** Increased Mettl3 mRNA expression was associated with poor overall survival (OS) in MM patients from TT2 cohort(p=0.0576). **g.** Increased HNRNPA2B1 mRNA expression was associated with poor overall survival (OS) in MM patients from TT2 cohort. **h.** Relationship between *HNRNPA2B1* expression and OS in MM patients from HOVON65 cohort. **i.** Doxycycline-induced shRNAs specifically resulted in decreased expression of *HNRNPA2B1* protein. Effect of *HNRNPA2B1* knockdown on MM cell proliferation. **j.** Myeloma xenografts in NOD-SCID mice (control, Left flank; *HNRNPA2B1* OE, Right flank). **k.** Time course of tumor growth in NOD-SCID mice (n=6). **l.** *HNRNPA2B1* overexpression affected the tumor weight. Tumor weight of the control and *HNRNPA2B1* OE groups at day 26 after injection of MM cells.

**Figure S2.** Identification of potential targets of *HNRNPA2B1* in MM cells via transcriptiome-wide m6A-seq assays. **a.** Venn diagram showed 60 genes m6A with over 2-fold expression change in sh*HNRNPA2B* compared with control in both ARP1 and H929 cells. **b.** Graphs of m6A peak distribution presented the proportion of total m6A peaks in the indicated regions in control and *HNRNPA2B1^KD^* cells (right). **c.** Number of m6A peaks identified in m6A-seq in control and *HNRNPA2B1^KD^* cells. **d.** Number of m6A-modified genes identified in m6A-seq. Common m6A genes contain at least 1 common m6A peak, while unique m6A genes contain no common m6A peaks.

**Figure S3.** HNRNPA2B1 regulates *ILF3* gene expression through m6A-dependent formation. **a.** Western blot analysis of ILF3 in MM cells transfected with sh*HNRNPA2B1*. **b.** *HNRNPA2B1* overexpression increased the expression of *ILF3* at mRNA level in MM cells. **c.** *HNRNPA2B1* overxpression resulted in the elevation of *ILF3* at protein level. **d,e.** MM cells were treated with cyclolencine at the concentration of 0 mM, 50 mM, 100 mM. The expression of *ILF3* was detected at RNA (**d**) and protein (**e**) levels in H929 cells after 48 h treatment. **f.** RT-qPCR following the addition of ActD was performed to detect *ILF3* mRNA stability in H929 cells. **g.** HNRNPA2B1 (red) localization was examined by confocal microscopy. (i) Nuclei were stained with DAPI (4′,6-diamidino-2-phenylindole, blue). Scale bar, 5 μm. (ii) Immunolocalization of HNRNPA2B1 in MM cells. (iii) Immunolocalization of ILF3. (iv) Merger of images of i, ii and iii, with the colocalized regions shown in orange.

**Figure S4.** *ILF3* is upregulated in MM and *ILF3* knockdown suppressed the growth of MM cells. **a.** Relationship between *ILF3* expression and OS of MM patients from TT2 cohort. **b.** Relationship between *ILF3* expression and OS of MM patients from HOVON65 cohort. **c.** Flow cytometry was used to detect apoptosis of MM cells. **d,e.** Western blot analysis of ILF3 and apoptosis markers in different MM cell lines. **f.** Confirmation of ILF3 knockdown in ARP1 and H929 cells under transfection with three independent siRNAs (Si1, Si2 and Si3). **g.** MTT results indicated that knockdown of ILF3 by Si1 reversed cellular proliferation induced by overexpression of HNRNPA2B1.

**Figure S5.** *HNRNPA2B1* promotes MM progression via enhancing of *ILF3*-mediated expression of *AKT3 in vitro*. **a.** RT-qPCR following the addition of ActD was used to detect *AKT3* mRNA stability in H929 cells. **b.** Enforced *HNRNPA2B1* expression affected the expression of *ILF3* and *AKT3* at mRNA level in ARP1 and H929 cells. **c.** Enforced *HNRNPA2B1* expression affected the expression of *ILF3* and *AKT3* at protein level in ARP1 and H929 cells.
