# Supplementary material for: HNRNPA2B1 promotes multiple myeloma progression by increasing AKT3 expression via m6A-dependent stabilization of ILF3 mRNA
Source: J Hematol Oncol. 2021 Apr 1;14:54. doi: 10.1186/s13045-021-01066-6 (PMC8017865; doi:10.1186/s13045-021-01066-6)
Supplement: Supplementary file 3 — Additional file 3. [file 13045_2021_1066_MOESM3_ESM.docx]

**Additional file 2**

**Materials and Methods**

***Cell culture***

Human multiple myeloma cell lines ARP1 and H929 from ATCC were cultured in RPMI 1640 Medium (BI, Israel) with 10% fetal bovine serum (BI, Israel) 100 U/mL penicillin and 100 mg/mL streptomycin (YEASEN, China). HEK293T cells were cultured in Dulbecco’s Modified Essential Medium (BI, Israel) with 10% ES-fetal bovine serum (BI, Israel) and 100 U/mL penicillin and 100 mg/mL streptomycin (YEASEN, China). All cells were cultured in a humidified atmosphere of 5 % CO_2_ at 37 ℃.

***Generation of stable cell lines knockdown/overexpressed HNRNPA2B1 and ILF3***

The *HNRNPA2B1* over-expression plasmid was purchased from TranSheep Bio-Tech Co., Ltd (Shanghai,China). The short hairpin RNAs (shRNAs) targeting site of human *HNRNPA2B1* mRNA sequence was designed by RNAi online design tools (TGCTGT

 TGACAGTGAGCGCGGAAATTATGGAAGTGGAAATTAGTGAAGCCACAGAT

GTAATTTCCACTTCCATAATTTCCTTGCCTACTGCCTCGGA) and synthesized by Genscript Co., Ltd (Nanjing, China). The oligonucleotides were inserted into pTRIPZ vector according to the instruction of manufacturer. The *ILF3*-shRNA (CCGGCCTTCCAAGATGCCCAAGAAACTCGAGTTTCTTGGGCATCTTGGAA

GGTTTTTG) was purchased from Genebay Co., Ltd (Nanjing, China). Lentiviral vectors were co-transfected into HEK293T cells with packaging vectors psPAX2 and pMD2.G using Liposomal Transfection Reagent (YEASEN, Shanghai). Lentivirus particles were harvested at 48 h after transfection and transduced into MM cells. And transduced MM cells were sorted with puromycin (2 μg/mL; MCE, USA). Doxycycline (Beyoyime, China) was used to induce shRNA expression.

***RNA interference***

Small interfering RNAs (siRNA) induced *AKT3* gene silencing by targeting *AKT3* mRNA and negative control RNAs (siNC) purchased from GenePharma Company

(Shanghai, China). Electroporation method was used to deliver siRNA into the cells using the BTX Gemini SC (USA) device in accordance with optimization of electroporation parameters. Western Blotting was performed to assess protein expression to ensure efficient knockdown.

***Western Blotting***

Cells were lysed in appropriate radioimmunoprecipitation assay (RIPA) buffer (0.1% SDS, 1% NP-40, 50 mM HEPES, pH 7.4, 2 mM EDTA, 100 mM NaCl, 5 mM sodium orthovanadate, 40 μM p-nitrophenyl phosphate) mixed with 1% Protease inhibitor cocktail (Pierce Biotechnology, Rockford, IL, USA) for 15 min on ice. Cell lysates were centrifuged at 12,000 rpm for 15 min at 4°C. The supernatant was collected and protein was quantified using a Bicinchoninic Acid (BCA) Protein Assay Kit (YEASEN, Shanghai). Proteins were separated in 12% SDS-PAGE gel and transferred to 0.45 μm PVDF membranes (Millipore, Billerica, MA, USA). 5% non-fat milk dissolved in TBST buffer was used to block the membranes for 1 h at room temperature. Then the membranes were incubated in the primary antibodies at 4°C overnight. Membranes were rinsed with TBST buffer and corresponding secondary antibodies incubated at room temperature for 1 h. Chemiluminescent detection was performed with the ECL kit (Thermo Fisher Scientifific, USA). Antibodies were as follows: HNRNPA2B1 (1:1000; Proteintech, China), ILF3 (1:1000; Proteintech, China), AKT3 (1:1000; Cell Signaling Technology; USA), β-Actin (1:1000; Proteintech, China), PARP (1:1000; Cell Signaling Technology, USA), Casepase 3 (1:1000; Cell Signaling Technology, USA), Cleaved Casepase 3 (1:1000; Cell Signaling Technology, USA).

***RT-qPCR***

Total RNA was isolated from MM cells using Trizol reagent (YEASEN, Shanghai).

500 ng of purified RNA was reverse transcribed using the Hifair 1st Strand cDNA Synthesis SuperMix for qPCR (gDNA digester plus) (YEASEN, Shanghai). PCR samples were prepared with diluted cDNA (1:30), 5 μL SYBR Green PCR master mix (YEASEN, Shanghai), 0.2 μM each of the forward and reverse primers in a total volume of 10 μL. GAPDH was used as internal standard. Quantitative PCR (qPCR) was performed using an Analytikjena qPCRsoft 4.0 (Gemany). The relative expression level of target gene was calculated using the 2^−ΔΔCT^ method and graphed as fold change (2^−ΔΔ^CT) from control.

Table S1 Primer Sequence

| Gene Name |  | Primer Sequence (5' -> 3') |
| --- | --- | --- |
| *GAPDH* | Forward: | GTCGGAGTCAACGGATT |
|  | Reverse: | AAGCTTCCCGTTCTCAG |
| *HNRNPA2B1* | Forward: | GGAGTGGAAGAGGAGGCAAC |
|  | Reverse: | CAGGTCCTCCTCCATACCCA |
| *ILF3* | Forward: | GCCCCCATCTTTACCATGTC |
|  | Reverse: | GCAAGCCCATGTCCTGTAAC |
| *AKT3* | Forward: | TGTGGATTTACCTTATCCCCTCA |
|  | Reverse: | GTTTGGCTTTGGTCGTTCTGT |

***Cell proliferation assay***

The viability of cells was detected using 3-[4, 5-di- methylthiazol-2-yl]−2, 5-diphenyltetrazolium bromide (MTT) assay. Cells were seeded into 96-well plates (5×10^3^ cells per well). Then, 20 μL of MTT reagent (5 mg/mL; Sigma-Aldrich, USA) was added into each well after incubated for 24, 48 or 72 h. The formazan was dissolved in DMSO and the absorbance was measured spectrophotometrically at 570 nm by a microplate reader (Thermo Fisher, USA) after incubated for 4 h.

***Cell apoptosis analysis***

Annexin V/PI staining assay was used to detect apoptosis of cells. The cells were harvested and re-suspended in 100 μL binding buffer and stained with 5 μL APC Annexin V (Biolegend, USA ) and Propidium Iodide (Solarbio, China) in darkness at room temperature for 15 min. Flow cytometry was performed by Guavaeasy Cyte (Merck Millipore, USA) and Annexin V positive cells were quantitated by FCM.

***MeRIP sequencing***

ARP1 and H929 cells with stable knockdown of HNRNPA2B1 for Methylated RNA immunoprecipitation (MeRIP) sequencing were stored in TRIzol reagent and MeRIP-Seq assay was carried out by LC Sciences (USA).

***RIP sequencing and RIP-qPCR***

An RIP experiment was performed using ARP1 cells according to the instructions of the article (Miriam Gagliardi and Maria R. Matarazzo. RIP: RNA Immunoprecipitation. Methods Mol Biol. 2016;1480:73-86). In brief, ~5-20 × 10^6^ cells were harvested and lysed. Protein A/G MagBeads pre-coated with 5 μg of the antibody of interest (HNRNPA2B1/ILF3, Proteintech) and incubated with cell lysate supernatant at 4°C overnight. The beads containing immunoprecipitated RNA-protein complex were treated with 150 μL of Proteinase K buffer to digest the proteins. Specific binding RNAs were isolated by using TRIzol and analyzed through RT-qPCR or highthroughput sequencing. Data analyses were performed as previously described. RIP-Seq assay was performed by Novogene (Beijing, China).

***RNA decay assay***

MM cells were treated with mRNA transcription inhibitor Actinomycin D (5 μg/mL) (MCE, HY-17559) for 0, 1, 2, 4, 6 h. Then, the total mRNA was isolated and followed by qRT-PCR to quantify the relative abundance of mRNA (relative to 0 h).

***Confocal immunofluorescent assay***

Cells were fixed with 4% paraformaldehyde in PBS at room temperature for 15 min then permeabilized in 0.5% Triton X 100 for 10 min and blocked with 4% BSA for 2 h at room temperature. The cells were incubated with primary antibody at 4°C overnight and subsequently fluorescent probe-linked secondary antibody incubated at 4°C for 2 h protected from light. HNRNPA2B1-flag blotted slides were incubated with Goat Anti-Mouse IgG (H+L) Fluor647-conjugated antibody (Affinity,USA )；ILF3 blotted slides were incubated with Goat Anti-Rabbit IgG H&L (Alexa Fluor 488) antibody (Abcam, UK). Nuclei were stained with 4’,6-diamidino-2- phenylindole (DAPI). All images were captured using confocal laser scanning confocal microscope (Leica, TCS SP8, Germany).

***Treatment of methylation inhibitors***

MM cells were exposed to cyclolencine (Sigma-Aldrich, USA) at the concentration of 0, 50, 100 mM for 48 h. RT-qPCR and western blotting analysis were followed to examine the expression of ILF3.

***Subcutaneous xenograft experiments***

The animal experiment protocol was approved by the Animal Ethics Committee of Nanjing University of Chinese Medicine (Ethics Registration no. 201905A003). 6-week SCID/NOD mice were purchased from Vital River Laboratory Animal Technology Co., Ltd. (Beijing, China).

Negative control and stably overexpressed HNRNPA2B1 MM cells were resuspended in PBS and injected subcutaneously into the left and right flanks respectively of SCID/NOD mice. The length and width of mouse tumor were measured every 2 days using a caliper. The tumor volume (TV) was calculated using the formula TV=（Length×Width^2）/2. At the end of the experiment, the mice were sacrificed and the tumor tissues were weighted.

### *Immunohistochemistry analysis (IHC)*

Immunostaining was performed on 3 µm paraffin tissue sections mounted on APES-coated slides. The main process was as follows: slides were incubated with the primary antibody overnight at 4°C. Afterwards, the secondary antibody was applied and kept for 45 min at 37°C, followed by dropping SABC at 37°C for 30 min, DAB coloring, and finally counterstaining with hematoxylin.

***Statistical analysis***

Statistical analyses were performed using SPSS version 22.0 or GraphPad Prism 6.01 software, and all values were expressed as mean ± SD unless otherwise specified. A two-tailed Student’s t-test (2 groups) or one-way analysis of variance (ANOVA) with Tukey’s posthoc comparison (≥3 groups) was utilized to evaluate statistical significance. A Kaplan–Meier curve and Log-rank test were employed to determine MM patient survival. p<0.05 was considered statistically significant.
